# ApoE4 Homozygosity Is Associated With Increased Microglia Activation in Fatal COVID‐19

**DOI:** 10.1111/neup.70033

**Published:** 2025-11-18

**Authors:** Amar Hamdan, Yassir El‐Amri, Fabian Heinrich, Osama A. A. Mohamed, Diego Sepulveda‐Falla, Markus Glatzel, Jakob Matschke, Susanne Krasemann

**Affiliations:** ^1^ Institute of Neuropathology University Medical Center Hamburg‐Eppendorf Hamburg Germany; ^2^ Institute of Legal Medicine University Medical Center Hamburg Eppendorf Hamburg Germany

**Keywords:** APOE, COVID‐19, microglia, neuroinflammation, nucleocapsid, SARS‐CoV‐2

## Abstract

Apolipoprotein E4 (APOE4) is recognized as a major risk factor for disorders such as Alzheimer's disease and may play a role in the dysregulation of microglia in various neurodegenerative conditions. APOE variants have also been proposed as potential risk factors for severe outcomes in COVID‐19. While fatal COVID‐19 may be associated with increased neuroinflammatory changes especially in the brainstem, it is not fully understood whether APOE4 contributes to the latter. In this study, we determined the APOE genotype from archival paraffin tissue blocks of 38 patients with fatal COVID‐19 who died either from infection with ancient SARS‐CoV‐2 or with the Omicron variant. Our data show an overrepresentation of APOE4 carriers during the first infection wave. Immunohistochemical staining of HLA‐DR/DQ/DP was performed to determine the abundance of activated microglia in different brain regions. This analysis displayed a correlation of APOE4/E4 with increased microglial activation in the cerebellum in fatal COVID‐19. Our data show a significant association between the APOE4 genotype and increased microglial activation, particularly affecting men who exhibit more microglial activity than women. In parallel, staining of SARS‐CoV‐2 nucleocapsid protein was used to assess the infection in the corresponding lung tissues. We found that a higher abundance of virus protein detection in the lungs was correlated with activation of microglia in the cerebellum in the latter cohort. Our work suggests that the cerebellum should be included when studying the impact of infectious diseases on the brain.

**Abbreviations**
AD: Alzheimer's dementia/disease
APOE: apolipoprotein E
CNS: central nervous system
COVID‐19: coronavirus disease 2019
DAB: 3,3'‐diaminobenzidin‐tetrahydrochlorid
FFPE: formalin‐fixed paraffin‐embedded
HLA: human leukocyte antigen
PASC: post‐acute sequelae of COVID‐19
PBS: phosphate‐buffered saline
PCR: polymerase chain reaction
SARS‐CoV‐2: severe acute respiratory syndrome coronavirus 2
WHO: World Health Organization

## 1 Introduction

The human apolipoprotein E (*APOE*) gene includes three common alleles that produce distinct protein isoforms: APOE2, APOE3, and APOE4, each with unique biological functions. In the central nervous system (CNS), APOE is mainly produced by astrocytes and is the main apolipoprotein responsible for regulating lipid metabolism [1, 2]. It is crucial for cholesterol recycling and redistribution in the brain, which affects membrane maintenance, organelle biogenesis, and synaptogenesis, essential for neuroplasticity [3].

Carriers of the *APOE4* allele have an increased risk for developing Alzheimer's dementia (AD) [4]. While APOE in the brain is mainly produced by astrocytes, recent studies have shown that particularly APOE4 also plays a role in the dysregulation of microglial cells in neurodegenerative diseases [5, 6, 7] and might contribute to the severity of these diseases. Microglia are the innate immune cells of the brain. Microglia play crucial roles in both healthy and diseased brains by regulating development, plasticity, and function. Additionally, microglia serve as the primary immune defense, providing protection against injury and infection in the brain. Homeostatic microglia change their morphology in response to external pathogens and other threats such as apoptotic cells and protein aggregates [8]. Microglial nodules are defined as focal aggregates of activated microglial cells within the brain parenchyma, frequently indicative of a localized neuroinflammatory response [9].

Interestingly, APOE4 has also been implicated as a risk factor for increased susceptibility to severe acute respiratory syndrome coronavirus 2 (SARS‐CoV‐2) and COVID‐19 mortality in human patients and mouse models [10, 11]. Moreover, the activation and dysregulation of microglia in patients with COVID‐19 have been shown in several studies [12, 13, 14, 15].

COVID‐19 is a viral respiratory tract illness with symptoms similar to those of the common cold, such as cough, fever, and runny nose. In contrast to other respiratory infections, more severe disease courses with fatal outcomes occur. COVID‐19 triggered a global pandemic since 2019, resulting in about 7 million official deaths (according to the WHO, as of July 2025) [16]. Moreover, the infection with SARS‐CoV‐2 can lead to acute and long‐term neurologic symptoms, such as concentration and memory disorders, fatigue, headaches, myalgia, and neuropathy. These symptoms may also play a role in post‐acute sequelae of COVID‐19 (PASC; long COVID) [17]. In this context, the cerebellum and brainstem represent important regions of interest in postmortem examinations of COVID‐19, given their central roles in motor coordination, autonomic regulation, and vital functions [12]. Although the cerebellum has been largely overlooked in COVID‐19 neuropathology studies, it may be especially susceptible in patients with COVID‐19 [18, 19, 20]. Diffuse parenchymal microglial activity and pronounced perivascular macrophage activation have been reported in the cerebellum and brainstem of COVID‐19 patients [20]. The cerebellum contains neuronal populations, such as Purkinje cells, that are highly sensitive to oxidative stress and pro‐inflammatory cytokines [21]. Moreover, the cerebellum exhibits robust expression of cytokine receptors (e.g., for IL‐6, TNF‐α, and other interleukins), and glial cells within this region—microglia as well as astrocytes—respond vigorously to immune challenges [21]. Increased microhemorrhages in the cerebellum in fatal COVID‐19 have been detected especially in APOE4‐positive patients [22].

Differences in the *APOE* genotype are significantly associated with survival in patients infected with SARS‐CoV‐2, based on candidate variant analysis in the UK Biobank cohort [10]. Moreover, Ostendorf et al. could show that experimental infection with SARS‐CoV‐2 of mice carrying the different human APOE isoforms led to increased lung pathology in carriers of APOE2 and APOE4 [10]. This was associated with a significantly reduced survival probability in APOE4 mice, which was further aggravated in male mice. However, the association of *APOE* genotype and abundance of SARS‐CoV‐2 in the lung of patients that died of COVID‐19 has never been investigated.

In our study, we determined the *APOE* genotype from archival paraffin blocks of patients who died of COVID‐19 in the first wave of infections in spring 2020 and of those who died in 2022 during Omicron (lineage B.1.1.529) abundance. Since the cerebellum and brainstem might be especially vulnerable in severe COVID‐19, we assessed microglial activation in dependence on the APOE genotype in fatal COVID‐19. We show here that patients carrying *APOE4/E4* display a significantly higher amount of microglia nodules and activated microglia than those with *APOE3/E4* or *APOE3/E3* especially in the cerebellum. Moreover, we analyzed the lung tissue of the respective patients for the abundance of virus‐protein positive cells. Interestingly, we detected a significant correlation between microglial activation in the cerebellum and the abundance of virus in the lung.

## 2 Materials and Methods

### 2.1 Human Tissue Samples—Disclosure of Ethical Statements

Autopsies of patients who had died following a diagnosis of SARS‐CoV‐2 infection were performed at the Institute of Legal Medicine of the University Medical Center Hamburg‐Eppendorf, Germany between March 13 and April 24, 2020 (SARS‐CoV‐2) and in January 2022 (Omicron lineage B.1.1.529). The use of human tissue paraffin blocks for scientific purposes after diagnostic procedures are concluded was reviewed and approved by the institutional review board of the independent ethics committee of the Hamburg Chamber of Physicians (protocol nos. PV7311, 2020‐10 353‐BO‐ff, and PV5034). The study is in line with the Declaration of Helsinki. Formalin‐fixed paraffin‐embedded archival frontal cortex, upper and lower medulla oblongata, and cerebellum brain tissues and lung tissues were used for this study. Patients were selected from the very first wave of COVID‐19 in Germany and from the first fatal cases of patients with Omicron, and for the availability of paraffin blocks from different brain regions and from lung tissue. A summary of patients included in this study is provided in Table S1.

A general overview of the neuropathological changes of the patients infected with the ancient SARS‐CoV‐2 had been published earlier in Matschke et al. 2020 [12], while a comparative study of the lung pathology of different variants of concern had been published in Heinrich and Huter et al. 2023 [18].

### 2.2 DNA Extraction From Paraffin‐Embedded Tissue

For the DNA extraction, we used formalin‐fixed paraffin‐embedded lung tissue. Initially, 2 mm tissue punches were made and deparaffinized overnight in a 70°C oven. This was followed by washing the tissue pieces with xylene three times for 30 min each. Subsequently, the samples were washed three times with 100% ethanol and three times with 70% ethanol, then washed twice with water. To each sample, 500 μL digestion buffer (1 M Tris–HCl, pH 8.0, 10% SDS, 0.5 M EDTA, pH 8.0, 5 M NaCl, and distilled water (dH₂O)) and 2 μL Proteinase K (Qiagen) were added, and the samples were incubated at 56°C overnight. The next day, the purification process continued using the Maxwell RSC FFPE Plus DNA‐Kit (Promega Corporation) according to the manufacturer. Briefly, 400 μL of lysis buffer was added to each sample, followed by centrifugation. New elution tubes were also prepared, each filled with 40 μL nuclease‐free water. These were placed in the Maxwell device. After the completion of the isolation process, the DNA concentration and purity were measured. Using this method, we obtained a ratio of DNA Purity of 1.8. DNA purity is typically evaluated using the absorbance ratio at 260/280 nm, with a ratio of ~1.8 generally indicating high‐purity DNA [19].

### 2.3 Determination of the *APOE* Genotype by qPCR

For the determination of the *APOE* genotype using DNA that was isolated from formalin‐fixed paraffin‐embedded tissue, a modified version of the method described by Calero et al. was used [23]. In this method, real‐time PCR is used with three primer sets each specific for one of the three human *APOE* haplotypes. The reaction originally included Power SYBR Green PCR Master Mix, primers at a concentration of 0.3 μM, and 50 ng of genomic DNA. However, using the DNA isolated from tissues in paraffin blocks, we had to optimize the DNA concentration. The clearest results were obtained, when the DNA concentration was adjusted to 20 ng/μl. Negative controls without DNA and positive controls with DNA isolated from cultured cells with known *APOE* genotypes including *APOE2*, *APOE3*, and *APOE4* were run in parallel. The amplification process consisted of an initial step at 95°C for 10 min to assure proper melting of double‐stranded DNA into single strands, followed by 40 cycles at 96°C for 35 s and at 63°C for 1 min for annealing/extension. The method was performed using the QuantStudio 5 Real‐Time PCR System for Human Identification and the QuantStudio 7 Flex Real‐Time PCR System (both from Applied Biosystems Thermo Fisher Scientific), utilizing the comparative Ct method for quantitation.

### 2.4 Immunohistochemical Staining

Formalin‐fixed, paraffin‐embedded (FFPE) brain tissue samples from the frontal cortex, upper and lower medulla oblongata, and cerebellar hemisphere were processed and stained using a Ventana Benchmark XT Autostainer (Ventana, Tucson, AZ, USA) according to the manufacturer's guidelines. Briefly, paraffin sections were cut and dewaxed. After inactivation of endogenous peroxidases (PBS/3% hydrogen peroxide), antibody‐specific antigen retrieval was performed. Sections were blocked and afterwards incubated with the primary antibody directed against human HLA‐DR (anti‐HLA‐DP/DQ/DR; Dako #M0775; 1:200) for staining. For detection of specific binding and DAB staining, the UltraView Universal DAB Detection Kit (Roche, #760‐500) was used. Counter staining and bluing were performed with Hematoxylin (Ventana Roche, # 760‐2021) and Bluing Reagent (Ventana Roche, # 760–2037).

Subsequently, microgliosis was classified as none (0), slight (1), moderate (2), or severe (3). The presence of microglial nodules using HLA‐DR as a marker of activated microglia was classified as none (0), 1–3 nodules (1), 4–10 nodules (2), or more than 10 nodules (3) per field of view/tissue. Both semi‐quantitative microglia staging was performed blinded to the genotype. For each case and brain region, 10 fields were analyzed using a 5× objective magnification (field of view ~12.57 mm^2^). In addition, a detailed analysis was performed at 10× objective magnification, with a corresponding field of view of approximately 3.14 mm^2^.

For the assessment of virus protein‐positive cells in the corresponding lung tissue sections, the anti‐SARS‐CoV‐2 nucleocapsid protein primary antibody (HS‐452011, Synaptic Systems, Göttingen, Germany; 1:1000) was used for detection. The abundance of SARS‐CoV‐2 positivity was quantified in a semi‐quantitative manner and was either scored as positive (virus protein‐positive cells detectable in the lung) or negative (no virus proteins detectable in the lung). The entire slide was scanned with a slide scanner (Nano Zimmer S20 MD, Hamamatsu) and representative pictures were selected.

### 2.5 Statistical Analyses

**FIGURE 1:**
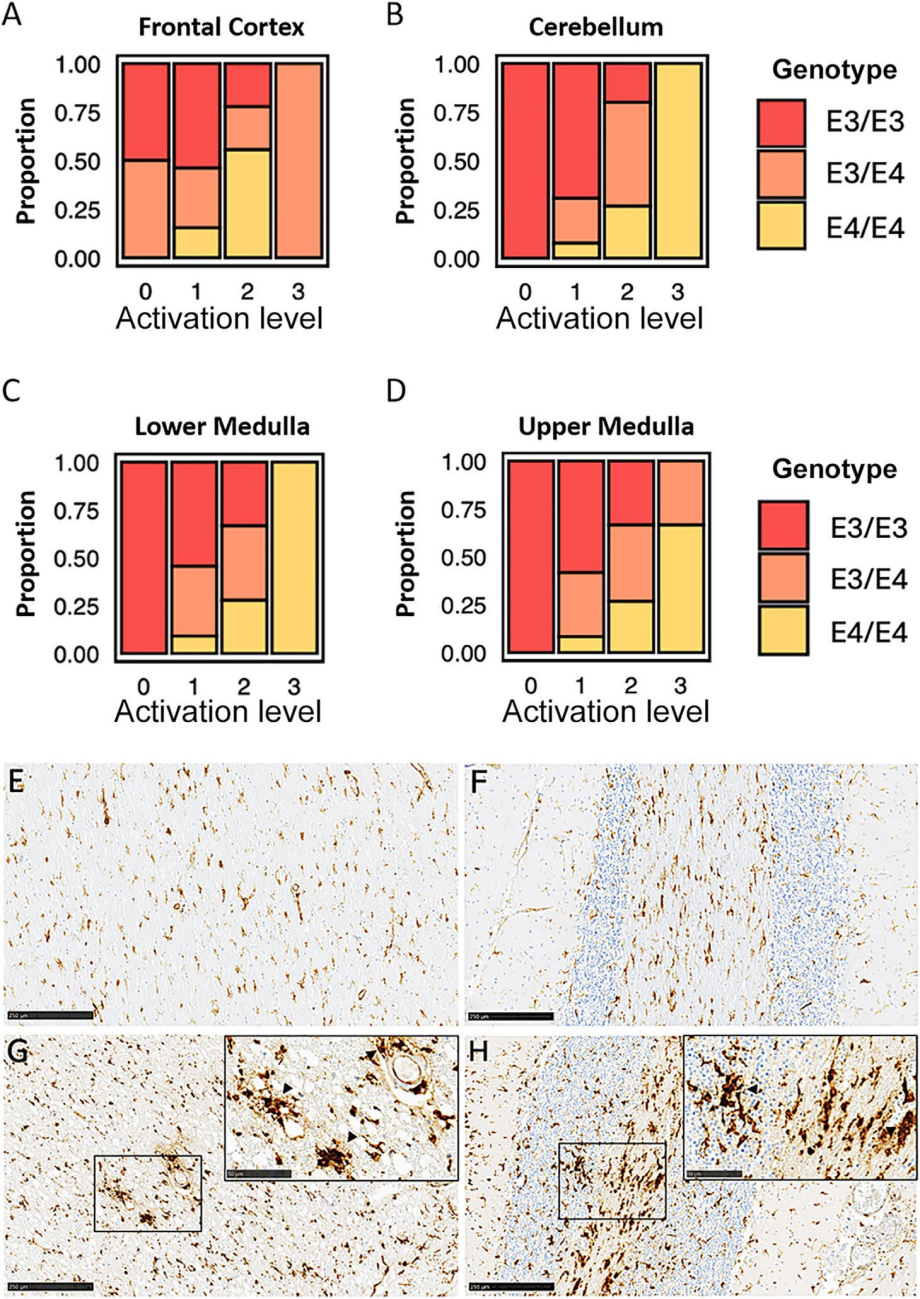
Microglia are highly activated in the cerebellum in homozygote *APOE4* carriers. Microglial activation in various brain regions, including (A) the frontal cortex (*p* = 0.0872), (B) cerebellum (*p* = 0.0036), (C) lower medulla (*p* = 0.3802), and (D) upper medulla (*p* = 0.3024) was measured on a semi‐quantitative scale from 0 (no activation) to 3 (high activation). The probability of activation in 32 cases is shown based on the APOE genotype. The y‐axis shows the proportion of microglia activation as a percentage, while the x‐axis displays the different brain regions analyzed. (E–H) Representative images of HLA‐DR/DQ/DP staining in cerebellum tissue are shown displaying the microglial activation status. (E–F) Representative HLA‐DR/DQ/DP staining of cerebellum tissue from two different *ApoE3/E3* carriers show low levels of microglial activation and no microglial nodules. (G–H) Representative CD68 staining of cerebellum tissue from two different *ApoE4/E4* carriers demonstrates strong microglial activation. Close‐up inserts show abundant microglial nodules (black arrow heads), located both perivascular and within the brain parenchyma. Scale bars: 250 μm; close‐up: 50 μm.

**FIGURE 2:**
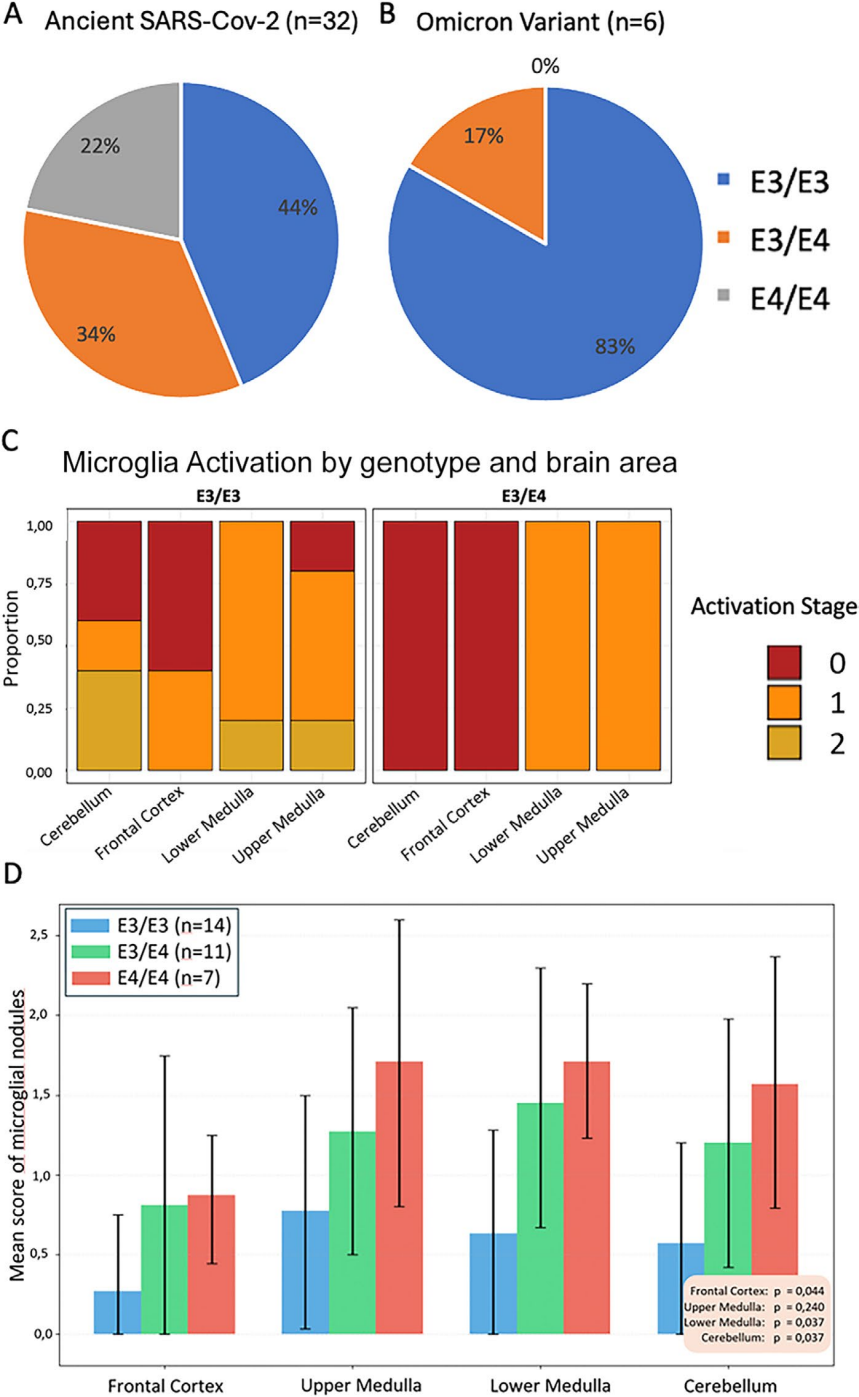
Overrepresentation of fatal COVID‐19 in early 2020 among APOE4 carriers. (A) Distribution of *APOE* genotypes in patients who died from COVID‐19 in early 2020. (B) Distribution of *APOE* genotypes in patients who died from the Omicron variant in early 2022. (C) The semi‐quantitative assessment of microglia activation in the brains of patients that died from the SARS‐COV‐2 Omicron variant revealed no or only very mild activation. The relationship between brain inflammation and specific brain regions categorized by *APOE* genotype is illustrated in patients infected with the Omicron variant (*n* = 6). The y‐axis shows the proportion of microglia activation as a percentage, while the x‐axis displays the different brain regions analyzed, such as the frontal cortex, medulla, and cerebellum. (D) Microglia nodules in different brain areas, including the frontal cortex, upper medulla, lower medulla, and cerebellum were categorized from 0 (without nodules), 1 (1–3 nodules), 2 (4–10 nodules), to 3 (more than 10 nodules) in patients who died of infection with the ancient SARS‐CoV‐2 (*n* = 32). The data represent the probability distribution of microglial nodule occurrence across 32 cases, stratified by APOE genotype and brain region. Fisher's exact test revealed significant correlations between microglial nodules and APOE4 genotype in the cerebellum (*p* = 0.0370), lower cortex (*p* = 0.0365), and frontal cortex (*p* = 0.0442), but not in the upper medulla (*p* = 0.2396).

**FIGURE 3:**
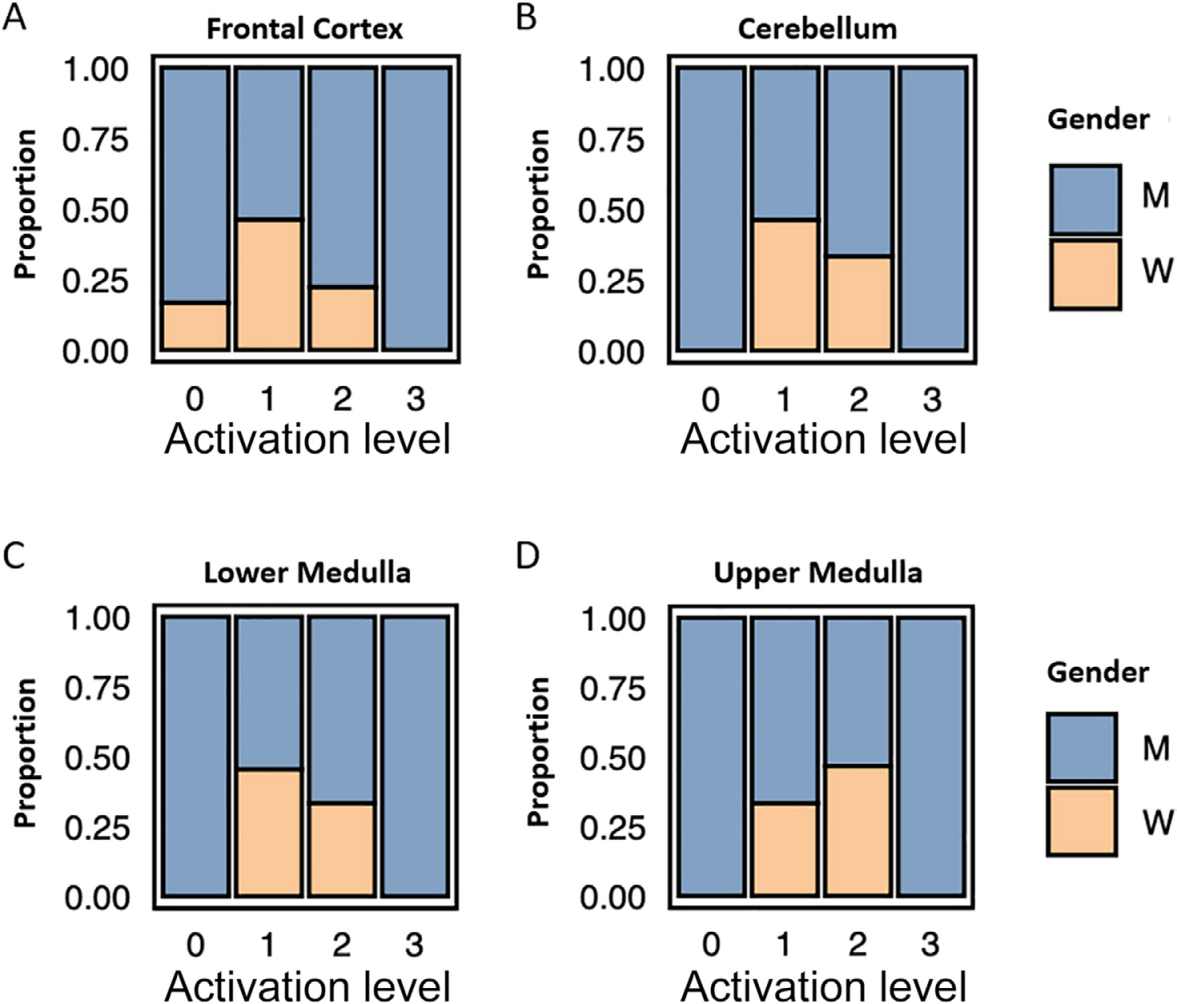
Microglia activation is more pronounced in patients with male sex. The analysis of microglial activation in the frontal cortex (A), cerebellum (B), lower medulla (C), and upper medulla (D) is shown after semi‐quantitative assessment of activation levels scored from 0 (no activation) to 3 (high activation). Graphs are based on probability distributions across 32 cases stratified by gender.

**FIGURE 4:**
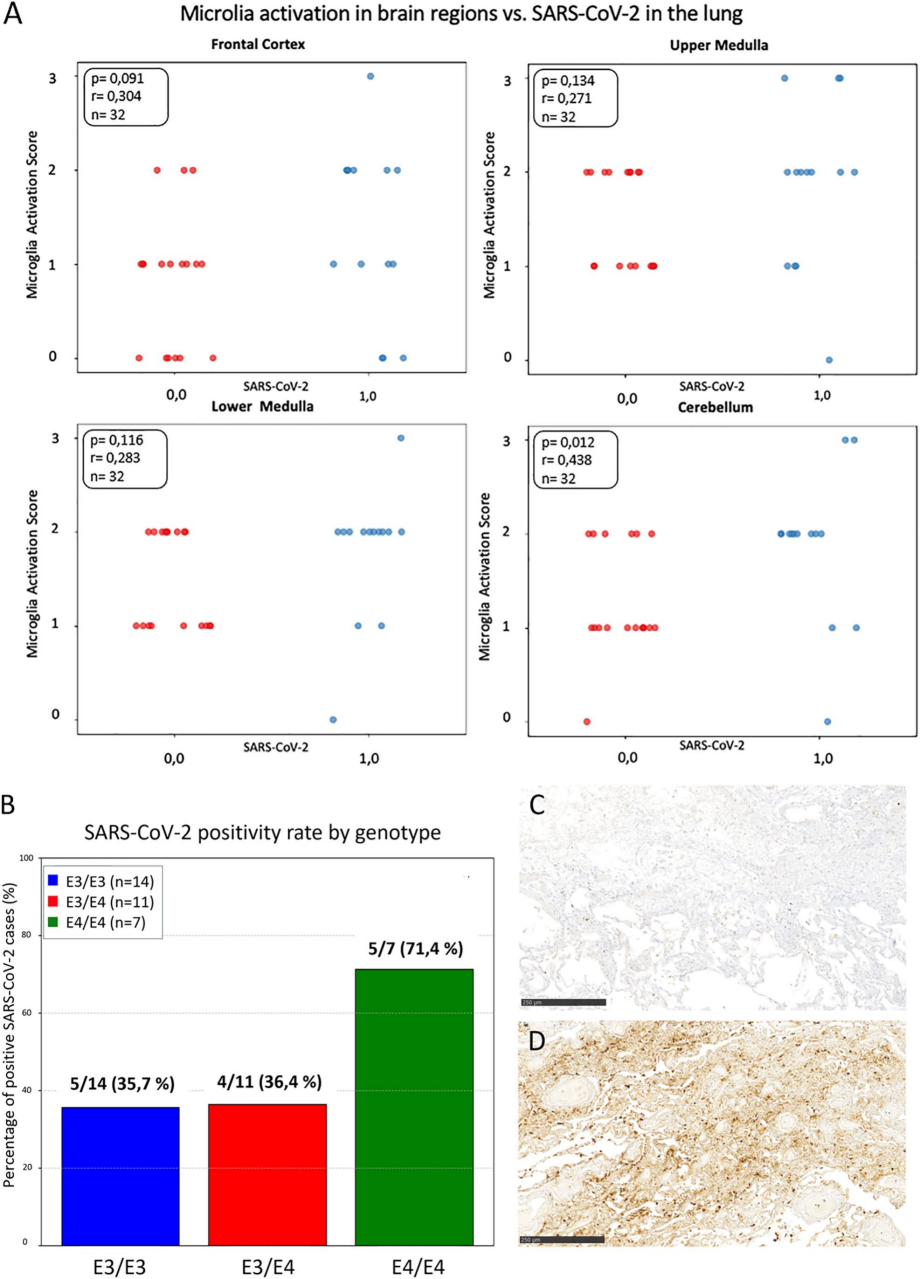
Abundance of SARS‐CoV‐2 nucleocapsid protein in the lung is associated with microglia activation in the cerebellum. (A) The correlation between microglial activation in different areas of the brain including the frontal cortex, upper medulla, lower medulla, and cerebellum, and abundance of SARS‐CoV‐2 nucleocapsid protein in the lung is shown. (B) Genotype‐dependent analysis of abundance of SARS‐CoV‐2 nucleocapsid protein in the lung showed increased viral presence in the lung in *APOE4* carriers (Chi^2^ *p* = 0.2478; Fisher's exact test: *p* = 0.1948). (C, D) Representative immuno‐histochemical staining of SARS‐CoV‐2 nucleocapsid protein in lung tissues of fatal COVID‐19 cases are shown. (C) In the majority of lung tissues from *APOE3* carriers, no SARS‐CoV‐2 nucleocapsid protein is detectable. (D) SARS‐CoV‐2 nucleocapsid protein is highly abundant within the lung of an APOE4 homozygote patient. Scale bar 250 μm.

Descriptive statistics were used to summarize patient demographics. The primary cohort included 32 COVID‐19 cases aged between 51 and 94 years (mean age: 77 years; 21 males, 11 females). A second Omicron cohort consisted of 6 cases aged between 49 and 99 years (mean age: 68 years; 5 males, 1 female). Age was summarized using the mean, and categorical variables were reported as counts. Statistical analyses and data visualization were conducted using R (R core team 2021) [24], Python (Python 3) [25] with the matplotlib.pyplot library and Microsoft Excel. A significance level of *p* < 0.05 was applied throughout. To evaluate associations between categorical variables—including APOE genotype and microglial activation, genotype and the presence of microglial nodules, and genotype and SARS‐CoV‐2 positivity in the lung—Fisher's exact tests were performed in Python using the matplotlib.pyplot library (Figures 1, 3, 4). For the association between genotype and SARS‐CoV‐2 presence, a Chi‐squared test was also applied (Figure 4). Spearman correlation analysis was used to assess the relationship between microglial activation levels and the presence of SARS‐CoV‐2 nucleocapsid‐positive cells in the lung. The results were visualized using jitter plots and scatter plots with regression lines in Python (matplotlib.pyplot) (Figure 4). Data visualization was performed in R using the ggplot2 package [26] to illustrate associations between microglial activation and APOE genotype. Microglial activation levels (0, 1, 2, 3) across various brain regions (Frontal Cortex, Upper Medulla, Lower Medulla and Cerebellum) were analyzed and visualized using stacked bar charts based on APOE genotype (Figures 1 and 2) and additionally analyzed in relation to gender (Figure 2). The distribution of genotypes (E3/E3, E3/E4, E4/E4) within the study population was created and visualized using Microsoft Excel with pie charts illustrating the relative proportions of each genotype group (Figure 2). The mean number of microglial nodules per region was compared across genotypes using bar charts (Figure 2). Group comparisons and SARS‐CoV‐2 positivity rates by genotype were visualized using bar plots in Python (matplotlib.pyplot) (Figure 2).

## 3 Results

### 3.1 Microglia Activation in Cerebellum Is Associated With *APOE4* Genotype

To investigate, if *APOE4* carriers that died from COVID‐19 in the first wave of infections in early 2020 show increased neuro‐inflammation, we determined the *APOE* genotype from archival tissue in paraffin blocks and assessed the microglia abundance and morphology using immune‐histochemical staining of HLA‐DR/DQ/DP. Microglial activation in the frontal cortex, upper medulla, lower medulla, and cerebellum, was assessed based on two criteria: the morphology of the microglia and the proportion of activated microglia in each section (Figure 1A–D). Semi‐quantitative analysis of microglial activation revealed a genotype‐dependent pattern, particularly pronounced in the lower medulla and the cerebellum. All cases classified as stage 0 for microglial activation in the upper medulla, lower medulla, and cerebellum, were from patients with APOE3/E3 genotype. In contrast, all individuals classified as Stage 3 in the lower medulla and cerebellum were identified as APOE4/E4 carriers. Although the overall sample size was limited (*n* = 32), statistical evaluation using the Fisher exact test demonstrated a significant association between *APOE4/E4* genotype and microglial activation in the cerebellum (*p* = 0,0036).

Representative immunohistochemical staining of HLA‐DR/DQ/DP in brain tissue from individuals with the APOE3/E3 genotype illustrates the low levels of microglial activation (Figure 1E,F). In these samples, microglia exhibit small, compact somata and thin, elongated, highly ramified processes—morphological features consistent with a non‐activated, more homeostatic state. In contrast, representative brain sections from individuals homozygous for the APOE4/E4 genotype display pronounced microglial activation (Figure 1G,H). Here, numerous microglial nodules are evident, both within the parenchyma and in perivascular regions (indicated by black arrows in the close‐up images of Figure 1G,H). Microglia in these regions exhibit hypertrophic morphology, with enlarged, rounded cell bodies and shortened, thickened processes that are less branched, indicative of an activated phenotype.

### 3.2 Overrepresentation of the *APOE4/E4* Genotype in Patients With Fatal COVID‐19 During the First Infection Wave

Genotypic analysis in 32 cases with fatal COVID‐19 after infection with the ancient SARS‐CoV‐2 revealed that 7 individuals (22%) were homozygous for the *APOE4* allele (*APOE4/E4*), 11 patients (34%) were heterozygous (*APOE3/E4*), and the remaining 14 cases (44%) were homozygous for the APOE3 allele (*APOE3/E3*) (Figure 2A). The frequency of *APOE4/E4* and *APOE3/E4* genotypes was higher than expected in the general population [27]. In contrast, the analysis of six fatal cases associated with infection by the Omicron variant showed a markedly different distribution: only one individual (17%) carried the *APOE3/E4* genotype, while the remaining five cases (83%) were *APOE3/E3* (Figure 2B). Notably, no *APOE4/E4* genotypes were observed in this group. Although based on a very low number of cases, these findings indicate a potential overrepresentation of the *APOE4/E4* genotype among early COVID‐19 fatalities during spring 2020, whereas its distribution appears more consistent with population norms in Omicron‐associated cases.

Microglial activation observed in patients infected with the Omicron variant was notably lower compared to levels documented during the initial waves of the COVID‐19 pandemic in 2020 (Figure 2C). Furthermore, no significant evidence for a correlation was detected between microglial activation and APOE genotype in the Omicron cohort. None of the investigated patients who died from the SARS‐CoV‐2 Omicron variant displayed highly activated microglia classified as Stage 3, but only showed moderate activation levels (Figure 2C). Although the small number of analyzed Omicron cases restricts conclusions, these findings may support the hypothesis that both heightened neuroinflammatory responses and the overrepresentation of the *APOE4/E4* genotype were specific to early pandemic cases.

### 3.3 Genotype‐Associated Microglia Nodules Are Abundant in Medulla and Cerebellum

We assessed the abundance of microglia nodules in a semi‐quantitative manner in the brain of patients who died of the ancient SARS‐CoV‐2 and analyzed their occurrence in correlation with the APOE genotype. The presence and regional distribution of microglial nodules demonstrated a genotype‐dependent pattern, with a higher frequency observed in individuals carrying the APOE4/E4 or APOE3/E4 alleles (Figure 2D). Using the Fisher exact test, significant correlations between microglial nodules and APOE4 genotype were identified in the cerebellum (*p* = 0.0370), lower medulla (*p* = 0.0365), and frontal cortex (*p* = 0.0442), whereas no significant association was detected in the upper medulla (*p* = 0.2396). Nodular microglial formations were most prominent in the lower medulla and cerebellum (see Figure 1H). In a subset of cases, these nodules were also observed in a perivascular distribution (see Figure 1G).

### 3.4 Increased Microglial Activation in Male Patients

The average age of patients in the primary COVID‐19 cohort was 77 years, with an age range of 50 to 94 years. Of these 32 patients, 21 were male (65.6%) and 11 were female (34.4%). Male sex is a known risk factor for severe and fatal COVID‐19 [10]. Thus, we also assessed the gender distribution in our cohort. When we investigated the degree of microglia activation in the different brain regions, we could show that male sex is associated with microglia activation (Figure 3A–D). Interestingly, none of the female patients displayed a stage 3 microglia activation.

### 3.5 Occurrence of Sepsis Does Not Correlate With APOE Genotype

In our cohort of 32 patients infected during the first wave, systemic inflammation, as reflected by the presence of sepsis, was observed in 14 individuals (43.75%) (Table S1). To explore potential associations, we applied Fisher's exact test to examine whether sepsis correlated with viral presence in the lung tissue as well as with different APOE genotypes (E3/E3, E3/E4, E4/E4). The analysis did not reveal statistically significant correlations, with a *p*‐value of 0.6592 for the association between APOE genotype and sepsis, and a *p*‐value of 0.4896 for the association between sepsis and SARS‐CoV‐2 in the lung. These findings suggest that, within our limited sample size, neither the presence of SARS‐CoV‐2 in the lungs nor the APOE genotype appears to be strongly associated with the development of sepsis. Notably, sepsis was not observed in patients infected with the Omicron variant, potentially indicating a reduced systemic inflammatory response. However, studies including large numbers of patients are needed to clarify potential genetic or viral contributions to systemic inflammatory responses in critically ill patients.

### 3.6 Microglial Activation Correlates With Detection of Virus Protein‐Positive Cells in the Lung in *APOE4/E4* Homozygotes

Mice carrying human APOE4 show an accelerated progression of disease and a higher number of SARS‐CoV‐2 nucleocapsid protein in the lung [10]. Thus, we analyzed the corresponding lung tissue of the here investigated patients for the abundance of SARS‐CoV‐2 nucleocapsid protein using immuno‐histochemistry. Microglial activation and pulmonary virus abundance were assessed in relation to the different brain regions (Figure 4A). A statistically significant correlation between microglial activation in the cerebellum and the viral burden in the lung was identified using the Spearman Test (*p* = 0.012). Furthermore, a proportional increase in pulmonary virus abundance was observed in individuals carrying the APOE4/E4 and E3/E4 alleles (Figure 4A), suggesting a potential link between APOE genotype and viral replication or clearance in the lung.

The SARS‐CoV‐2 detection in lung tissue—categorized as virus‐positive or virus‐negative—is presented across different *APOE* genotypes (Figure 4B). Individuals with the *APOE3/E3* genotype exhibited the highest proportion of virus protein‐negative lung samples. Patients carrying the *APOE3/E4* genotype demonstrated an increase in virus‐positive cases relative to the *APOE3/E3* group. Notably, individuals homozygous for *APOE4* showed a predominance of virus‐positive lung tissue over virus‐negative cases (Figure 4B). No significant association was observed between the APOE4 genotype and SARS‐CoV‐2 presence in the lung, as determined by Fisher's exact test (*p* = 0.1948) and the chi‐square test (*p* = 0.2478).

Virus‐negative cases showed no detectable evidence of viral presence (Figure 4C), whereas in SARS‐CoV‐2 nucleocapsid‐protein positive cases, a high number of infected cells was observed in our study (Figure 4D). Among the fatal six cases that were infected with the Omicron variant, histological analysis revealed that none showed detectable SARS‐CoV‐2 nucleocapsid protein in the corresponding lung tissues.

## 4 Discussion

The COVID‐19 pandemic, caused by the novel coronavirus SARS‐CoV‐2, has led to significant global morbidity and mortality. However, individual disease outcomes are highly heterogeneous and have been influenced by factors such as viral variants, healthcare capacity, vaccination status, and host genetic predisposition [10, 28, 29, 30]. Among the latter, the *APOE* genotype, specifically *APOE4*, has been implicated in impacting mortality and increasing the risk for severe COVID‐19 outcomes [10, 11]. *APOE4* is a well‐established genetic risk factor for late‐onset AD and has been shown to influence the degree of microglia activation and neuroinflammation in the context of neurodegenerative diseases [27, 31]. Neuroinflammatory changes have also been frequently reported in fatal COVID‐19 [12, 13, 32].

Our data supports an *APOE* genotype‐dependent pattern of microglial activation in patients who died from COVID‐19 during the first wave of the pandemic. Moreover, our findings show that the APOE4 genotype, particularly in individuals with the homozygous APOE4/E4 variant, is strongly associated with increased microglial activation in the cerebellum. Interestingly, Kurki et al. reported elevated presence of microvascular hemorrhages specifically in the cerebellum in APOE4 carriers in fatal COVID‐19 [22]. In contrast to our study, they did not detect APOE genotype‐dependent changes in the number of activated microglia in their cohort, but only studied midbrain, pons, and medulla, and did not stratify for *APOE4* homozygote patients. In our study, high levels of microglial activation in APOE4/E4 carriers were also observed in the upper and lower medulla; however, statistical significance was only reached for the cerebellum. The cerebellum and medulla are regions implicated in brainstem autonomic regulation and respiratory control. The vagus nerve is an essential component of the autonomic nervous system, transmitting sensory information from the lungs to the brain and back. We and others could recently show that inflammation of the vagus nerve with inflammatory alterations in the connecting brain areas might contribute to dysautonomia in severe COVID‐19 [33, 34]. Interestingly, proteomic and transcriptomic analyses reveal that local CNS reactions in the brainstem and cerebellum are attributed to systemic inflammation after severe respiratory infection without detection of SARS‐CoV‐2 in the brain [33].

A possible mechanism underlying cerebellar involvement in APOE4 carriers may be that, although the cerebellum shows limited amyloid‐β and tau pathology in early stages, its resident glial cells, microglia, and astrocytes, can respond to hippocampal pathology either through altered network activity or systemic inflammatory signals [35, 36]. Microglial activation in the cerebellum may therefore mediate the impact of APOE4‐driven hippocampal pathology on cerebro‐cerebellar functional connectivity, contributing to early network alterations and potentially influencing cognitive performance even before overt cerebellar pathology emerges [36].

A potential explanation for the heightened cerebellar microglial activation observed in APOE4/E4 carriers comes from insights into cerebellar microglial biology. Kana et al. demonstrated that cerebellar microglia possess a unique molecular identity, distinct from forebrain microglia [37]. Disruption of CSF‐1 in mice led to severe depletion and transcriptional alterations of cerebellar microglia, accompanied by reduced Purkinje cells, impaired neuronal function, and deficits in motor learning and social behaviors. These findings highlight the high density and functional importance of cerebellar microglia, suggesting that they are particularly sensitive to metabolic and inflammatory disturbances [37, 38].

Interestingly, the immunohistochemical analysis of corresponding postmortem lung tissues in our study revealed that APOE4/E4 individuals were more likely to have high SARS‐CoV‐2 nucleocapsid protein abundance in the lung. The latter could be due to a higher pulmonary viral burden, impaired viral clearance, or altered disease kinetics. These findings align with experimental studies in humanized mice. Ostendorf et al. demonstrated that *APOE4*‐targeted replacement mice exhibited significantly higher levels of SARS‐CoV‐2 nucleocapsid protein in the lungs and experienced more severe disease progression compared to *APOE3* counterparts [10]. These mice also showed enhanced inflammation. We assessed sepsis as a proxy for systemic inflammation in our patients and could not detect a correlation with APOE4 genotype. However, since bacterial superinfection could also lead to sepsis, this parameter is not very reliable. In a very recent study, Shvetcov et al. investigated the proteomic profile in cerebrospinal fluid and plasma from patients across different neurodegenerative diseases [39]. Interestingly, APOE4 carriers share systemic proteomic changes of plasma proteins that are indicative of a dysregulated immune system. Thus, APOE4 may be a broader modulator of systemic immunity, creating a vulnerability to disease‐specific factors.

Although the sample size in this study is modest (*n* = 32), the findings presented here suggest a potential overrepresentation of the *APOE4/E4* genotype among individuals who succumbed to COVID‐19 during the first wave of the pandemic in early 2020. Here, APOE4/E4 homozygosity was observed in 22% of cases—substantially higher than the estimated population frequency of ~2%–3% in most European populations [27, 40]. This enrichment, alongside the elevated frequency of the APOE3/E4 genotype, supports prior studies indicating that carriers of the E4 allele of APOE are at increased risk for severe COVID‐19 outcomes, including hospitalization, neurological complications, and mortality [10, 41]. In contrast, other studies have not seen such associations with disease severity [42]. This might be due to shifting patterns between the first wave and later time points, since the latter study included patients who were monitored between November 2020 and June 2021. This is in line with our data on the analysis of fatal COVID‐19 cases associated with the Omicron variant that revealed no APOE4/E4 individuals, and only a minority carried the APOE3/E4 genotype. In those cases, we could likewise not detect SARS‐CoV‐2‐positive cells in the corresponding lung tissue. The fatal cases from the first pandemic wave investigated in our study showed a high number of comorbidities. As noted later as a risk factor for severe COVID‐19, we also report an excess in male patients in our study [43, 44]. In contrast, women have a higher risk of developing ad [ 45]. Differences and specific vulnerabilities between the two groups—first wave versus Omicron—may also have been influenced by other factors. Interestingly, the here investigated fatal Omicron cases had a high number of comorbidities that were comparable to the first wave cases in our study. However, the introduction of anti‐SARS‐CoV‐2 vaccines has been a game changer in the fight against the pandemic and is estimated to have saved millions of lives [46]. Thus, the availability of vaccines may also have influenced patients' vulnerability during Omicron.

Beside direct APOE4‐related effects on COVID‐19 severity, patients homozygous for APOE4 might also be more vulnerable due to APOE4‐dependent co‐morbidities such as late‐stage dementia. APOE4 has also been shown to affect blood–brain barrier (BBB) function [47]. Moreover, microglia might be intrinsically dysregulated by APOE4 [48, 49]. Interestingly, we did not observe APOE genotype‐dependent effects on microglia activation or abundance of microglial nodules in the frontal cortex. Thus, the here described APOE4‐dependent effects on microglia activation in the cerebellum might be specific for COVID‐19; however, these findings are based on a limited sample of only 38 cases, which may restrict the generalizability of the results. Mechanistic studies on the role of APOE4 in COVID‐19 indeed show a differential effect that is independent of the dementia risk. Wang et al. reported an increased rate of SARS‐CoV‐2 infection in iPSC‐derived APOE4 neuronal cells and organoids [27]. Moreover, APOE4‐positive cells of the BBB such as endothelial cells and pericytes might also be more vulnerable to infection with SARS‐CoV‐2 [50].

The impact of APOE4 on long‐term disease outcome is still unclear. Kurki et al. reported higher mental fatigue in APOE4 carriers even 6 months post‐infection [22]. In contrast, Fernandez‐de‐Las‐Penas could not associate APOE4 with an increase in long COVID symptoms [51]. Interestingly, ApoE4 was shown to negatively affect the severity of breakthrough infections after COVID‐19 vaccination [52].

A limitation of this study is the imbalance between cases from the first wave of the pandemic (*n* = 32) and those from the Omicron period (*n* = 6). The very limited size of the Omicron cohort reduces the reliability of conclusions regarding potential differences in the role of the APOE4 genotype across pandemic phases. As such, these findings should be considered exploratory and interpreted with caution. Notably, none of the six Omicron cases carried the APOE4/4 genotype. While this might suggest a lower prevalence of APOE4/4 among Omicron cases, it could reflect the result of the very small cohort size, and no conclusions can be drawn.

Future studies using larger cohorts, but also comparing more systematically early pandemic cases, later cases, and those with current variants of concern, combined with techniques such as single‐cell transcriptomic analyses could further elucidate the mechanisms by which APOE4 could modulate immune activation and disease severity in viral infections. Additionally, the inclusion of data from other cortical regions, as well as from the basal ganglia and limbic system, would allow a more comprehensive evaluation of COVID‐19–related sequelae affecting cognitive function.

## Ethics Statement

Approval of the research protocol: The use of human tissue paraffin blocks for scientific purposes after diagnostic procedures are concluded was reviewed and approved by the institutional review board of the independent ethics committee of the Hamburg Chamber of Physicians (protocol nos. PV7311, 2020‐10 353‐BO‐ff, and PV5034). The study is in line with the Declaration of Helsinki.

## Consent

The authors have nothing to report.

## Conflicts of Interest

The authors declare no conflicts of interest.

## Supporting information


**Table S1:** Summary of cases including brain and lung autopsy findings.

## Data Availability

The data that support the findings of this study is available in the Supporting Information of this article.
