# Supplementary material for: ApoE4 Homozygosity Is Associated With Increased Microglia Activation in Fatal COVID‐19
Source: Neuropathology. 2025 Nov 18;45(6):e70033. doi: 10.1111/neup.70033 (PMC12626732; doi:10.1111/neup.70033)
Supplement: Supplementary file 1 — Table S1: Summary of cases including brain and lung autopsy findings. [file NEUP-45-0-s001.docx]

| **Case Number** | **gender** | **age range** | **cause of death** | **Sepsis** | **Comorbidities Number** | **neurological disorders** | **brain weight, g** | **brain atrophy** | **Macroscopic findings** | **SARS-CoV-2 in the lung** | **ApoE**  **genotype** |
| --- | --- | --- | --- | --- | --- | --- | --- | --- | --- | --- | --- |
| 1 | W | 50-54 | pneumonia | No | 1 | No | 1470 | None | None | neg | E3/E3 |
| 2 | W | 85-89 | Sepsis, pneumonia | Yes | 3 | No | 1310 | None | None | pos | E4/E4 |
| 3 | M | 70-74 | Pulmonary arterial embolism, pneumonia | Yes | 3 | No | 1665 | None | None | neg | E3/E4 |
| 4 | W | 80-84 | Pneumonia | Yes | 3 | No | 1290 | Mild | Mild | neg | E3/E3 |
| 5 | W | 90-94 | Sepsis | Yes | 6 | Dementia, history of stroke | 1220 | Mild | Old infarction in territory of PCA | pos | E3/E4 |
| 6 | M | 85-89 | Pneumonia | No | 6 | No | 1290 | None | None | neg | E3/E4 |
| 7 | M | 75-79 | Sudden cardiac death | Yes | 1 | PD | 1110 | None | None | neg | E3/E3 |
| 8 | M | 65-69 | Pneumonia | No | 2 | No | 1450 | None | None | neg | E3/E4 |
| 9 | W | 75-79 | Pneumonia | No | 2 | No | 1210 | Mild | None | neg | E3/E3 |
| 10 | M | 60-64 | Pulmonary arterial embolism, pneumonia | No | 1 | No | 1435 | None | Fresh infarction in territory of ACA | neg | E3/E4 |
| 11 | M | 85-89 | Pneumonia | Yes | 3 | PD | 1170 | Mild | Old infarctions in territory of PCA | neg | E3/E3 |
| 12 | W | 80-84 | Purulent bronchitis | No | 3 | No | 1080 | Mild | None | pos | E3/E3 |
| 13 | M | 80-84 | Pneumonia, septic encephalopathy | Yes | 5 | No | 1350 | None | None | pos | E3/E4 |
| 14 | M | 75-79 | Pulmonary arterial embolism, respiratory tract infection | No | 2 | No | 1460 | None | None | pos | E3/E3 |
| 15 | M | 85-89 | Pneumonia | No | 5 | No | 1540 | None | Cerebellar metastasis of non-small cell lung cancer | pos | E4/E4 |
| 16 | W | 75-79 | Pulmonary arterial embolism | No | 2 | No | 1095 | Moderate | None | pos | E3/E3 |
| 17 | M | 90-94 | Pneumonia | Yes | 5 | No | 1000 | Moderate | Old cerebellar infarction | pos | E3/E3 |
| 18 | M | 85-89 | Sepsis, pneumonia | Yes | 5 | epilepsy, hypoxic brain damage | 1210 | Mild | None | pos | E4/E4 |
| 19 | W | 85-89 | Pneumonia | Yes | 2 | No | 1180 | Mild | None | neg | E3/E3 |
| 20 | M | 50-54 | Pneumonia | No | 1 | No | 1255 | Mild | None | pos | E3/E3 |
| 21 | M | 55-59 | Pneumonia | No | 5 | No | 1230 | None | Old infarctions in territory of PCA and lenticulostriate arteries | pos | E4/E4 |
| 22 | W | 75-79 | Pneumonia | No | 2 | No | 1180 | Mild | None | neg | E4/E4 |
| 23 | M | 60-64 | Pneumonia | Yes | 1 | No | 1575 | None | None | neg | E3/E3 |
| 24 | M | 90-94 | Pneumonia | No | 2 | No | 1400 | None | None | pos | E3/E4 |
| 25 | M | 90-94 | Pneumonia | Yes | 4 | Dementia, history of stroke | 1015 | Moderate | Old infarctions in territory of PCA | neg | E3/E4 |
| 26 | W | 85-89 | Pneumonia | Yes | 3 | No | 1490 | None | Fresh infarction in territory of MCA | neg | E3/E3 |
| 27 | M | 70-74 | Pneumonia (aspiration) | No | 4 | PD | 1430 | None | None | pos | E3/E4 |
| 28 | M | 85-89 | Pneumonia | No | 4 | No | 1400 | None | None | neg | E3/E4 |
| 29 | M | 70-74 | Sudden cardiac death | No | 3 | No | 1430 | None | None | pos | E4/E4 |
| 30 | W | 70-74 | Pneumonia | Yes | 1 | No | 1150 | Mild | None | neg | E3/E3 |
| 31 | M | 90-94 | Emphysema with respiratory decompensation | No | 2 | No | 1440 | Mild | None | neg | E4/E4 |
| 32 | M | 75-79 | Pneumonia | No | 5 | No | 1590 | None | None | neg | E3/E4 |

**Abbreviations:**
neg = negative, pos = positive, PD = Parkinson’s disease, w = women, m = men, ApoE = apolipoprotein E.

**Omicron Cases**

| **Case** | **gender** | **Age range** | **cause of death** | **Sepsis** | **Comorbidities Number** | **Neurological disorders** | | **brain weight, g** | | **brain atrophy** | | **Macroscopic findings** | **SARS-CoV-2 in the lung** | | **ApoE**  **genotype** |
| --- | --- | --- | --- | --- | --- | --- | --- | --- | --- | --- | --- | --- | --- | --- | --- |
| 1 | w | 95-99 | decompensated heart insufficiency | No | 3 | yes (Alzheimer´s) | 1030 | | moderate | | none | | neg | E3/E3 | |
| 2 | m | 45-49 | aspiration due to gastrointestinal bleeding | No | 4 | No | 1500 | | mild | | none | | neg | E3/E3 | |
| 3 | m | 75-79 | aspiration of foreign material (bolus death) | No | 2 | yes (FTLD-TDP43) | 1390 | | mild | | none | | neg | E3/E3 | |
| 4 | m | 75-79 | COVID-19 pneumonia | No | 4 | NO | 1360 | | mild | | none | | neg | E3/E3 | |
| 5 | m | 55-59 | decompensated heart insufficiency | No | 2 | unkown | 1385 | | none | | subacute media infarction | | neg | E3/E4 | |
| 6 | m | 50-54 | pneumonia with pleural empyema | No | 3 | unkown | 1420 | | none | | atrophy of superior cerebellar vermis | | neg | E3/E3 | |

**Abbreviations:**
neg = negative, pos = positive, PD = Parkinson’s disease, w = women, m = men, ApoE = apolipoprotein E, FTLD-TDP43: Frontotemporal Lobar Degeneration with TDP-43 Pathology.

 Supplementary Table 1: **Summary of cases including brain and lung autopsy findings**
